# Pensions for Old Age

**Published:** 1893-03-18

**Authors:** 


### Pensions for Old Age.

### II.—ESSENTIAL POINTS.

In a former article some attempt was made to examine the proposals of the Post Office, of Canon Blackley, of Mr. Chamberlain, and of Mr. Charles Booth to solve the question of providing an old age pension for the people. It was seen that the Post Office has entirely failed to attract even those willing and able to insure for small amounts. It was seen also that grave objections attached to the proposals of Canon Blackley and Mr. Booth, while Mr. Chamberlain's scheme can only reach the comparatively prosperous, who least need the assistance.

But it is certain that no honest attempt to grapple with a a difficult question is ever entirely fruitless.

Canon Blackley has done incalculable service as pioneer of this great movement in emphasizing the necessity for compulsion in any measure which is to embrace the whole extent of old age misery. It is one thing to make it possible for the prosperous and prudent to provide against spending their closing years in poverty; it is quite another matter to make this duty imperative upon the ignorant, the idle, and the recklessly extravagant. Yet unless the pension is to be a free gift to all, the latter course alone will meet the existing need. Now the main stumbling-blocks in the way of all attempts at enforcing compulsory insurance are, first, the great expense (amounting often to ten per cent. of the total) attaching to the collection of contributions, and secondly, the extreme difficulty of procuring any contributions at all from the irregular wage earner, who forms a large proportion of all town populations. The machinery which must be put in motion to collect and credit to the person insuring, an infinitude of small amounts may well dismay the organizer of even a partial scheme. In Germany, where these difficulties have been boldly grappled with, a new forest of officials has sprung up and a network of regulations and penalties entangles the unwary. Every one must feel that this is intolerable. The German system, moreover, by which the employer is charged with the duty of deducting the contributions of the workman from his weekly wage, and adding to it a corresponding amount, could never take root in England where it would be regarded with suspicion as certain to hamper further the already complicated relations between labour and capital.

Three points must be found in any scheme which is to command general confidence; simplicity, universality, and justice to all classes. The first two are present in Mr. Booth's scheme alone and it has seemed as if the third point was wholly irreconcileable with either of them. Yet the problem has become narrowed to a very small issue. According to Mr. Sutton's tables, based on the most cautious actuarial calculations, the sum of 15s. yearly paid from the age of 18 should produce, at 2½ per cent. compound interest (money not returnable in case of death), a weekly annuity of 5s. at the age of 65.

Now 15s. a year is almost exactly 3½d. a week or one halfpenny a day. If this amount could be contributed by every person in the Kingdom between the ages of 18 and 65, the pension question would be solved. But would it be possible to ensure the contribution of so vast an amount of small sums? It does not sound a very easy matter, but it must be remembered that a very similar collection is actually carried out, by which every individual not State-supported is made to contribute his share towards the expenses of the nation and the advantages of civilization. The man who pays 4s. a week for the one room in which he and his family are huddled together, can reckon that at least a fourth part of that sum is absorbed for his landlord's rates and taxes. Even the casual loafer who gets his night's lodging for 3d. does not escape his fair proportion; his name is not entered as a ratepayer, but there is no question from whose pocket the amount ultimately paid is drawn.

Now, if the existing machinery for the collection of taxes were adopted to include an old age insurance, the contributors would fall into three broad classes.

For the middle and upper classes a quarterly return would be sufficient, and the addition of 3s. 9d. per adult head to the quarter's rent would not be a matter of great moment. Each householder would be required to fill in a return stating merely the number of adults sleeping under his roof during the past three months, and would be responsible for the sum total. The amount would be deducted from the wages of domestic servants.

The second-class of payments would be made by the large class of persons who have a weekly tenancy of their rooms or houses. Here the rent is collected weekly by the landlord or his agent from a constantly varying population. The landlord or rent collector would be made responsible for the sum total for the house weekly which might vary slightly according to the description of the tenants. The additional 3½d. (or 7d. in the case of married couples) would be felt no doubt by the occupier as something of a burden in many cases where the earnings are irregular. Yet it is just this class upon whom the burden of aged parents falls most heavily, and the family finances would often be greatly relieved by the payment of the weekly pension for one of the old people. It is believed that many of the most improvident would be thankful to be compelled to make provision for the future. A special form of collection would be necessary in country districts where the labourer pays his rent quarterly or has it assigned to him rent free in consideration of his services.

Hotel and lodging-house keepers would fall also under this second class; they would be responsible weekly for the total of adult visitors, and the amount would speedily become as familiar an item in the bill as the Continental "bougie."

Descending a step lower we come to the third class, viz., that portion of the town population which subsists by casual labour, has no settled home, and obtains a night's lodging by payments varying from twopence to sixpence. Even for this class the halfpenny toll on the night's lodging would not be an unreasonable or impossible demand. The recklessness as regards money of the very poor is proverbial, but a compulsory saving of even a halfpenny a day, if secured regularly as a preliminary to the night's lodging, might do much to foster a sense of thrift even among those whom we have been systematically pauperizing for generations.

A certain proportion of the population would, no doubt, escape payment. Hospitals, asylums, and certain benevolent institutions must be exempt. Inmates of the workhouses and casual wards, too, would be paying nothing. But not many except the aged find a permanent refuge in the workhouse, nor do many persons use the casual wards as a regular convenience. The loss would not be appreciable. It would be assumed without any attempt at registration that all persons reaching the age of 65 had entitled themselves by payment to a pension, and it is believed that except in cases of exceptional distress, the payment could and would be made.

The payments have been calculated on the basis that no money will be returned to the survivors in case of premature death. The low value in which life is held in certain quarters, has been only too conclusively shown by the experience of the burial societies, and any wide-spread system by which death would bring money compensation to the survivors would certainly entail grievous risk of crime.

The proportion of husbands and wives who reach old age together is so small that it is a question whether some reduction might not be made in the insurance of married couples. This and many other details would need careful actuarial consideration.

Thrift is a habit far more than a virtue, and the educational value of compulsory saving can hardly be overestimated. The bugbear of state interference is losing many of its terrors on a nearer acquaintance. Acts of Parliament have been found to be powerful agents when directed against ignorance, cruelty, disease, and dirt. Why should we shrink from directing the same force against improvidence? The vast organization necessary to undertake this vast work belongs to the state alone. It can hardly be doubted that sooner or later the responsibility of the nation will be felt and accepted towards the wellbeing of those among us who have borne to such good purpose "the burden and heat of the day."

